# DNA Methylation and Metabolic Syndrome in Midlife Women of the ELEMENT Cohort

**DOI:** 10.3390/epigenomes10040060

**Published:** 2026-09-24

**Authors:** Jiahe Tian, Jaclyn M. Goodrich, Lindsey English, Dana C. Dolinoy, Erica C. Jansen, Peter X. K. Song, Libni A. Torres-Olascoaga, Martha M. Téllez-Rojo, Karen E. Peterson

**Affiliations:** 1 Department of Environmental Health Sciences, University of Michigan School of Public Health, 1415 Washington Heights, Ann Arbor, MI 48109, USA; jhtian@umich.edu (J.T.); ddolinoy@umich.edu (D.C.D.); karenep@umich.edu (K.E.P.); 2 Department of Nutritional Sciences, University of Michigan School of Public Health, Ann Arbor, MI 48109, USA; lindseae@umich.edu (L.E.); janerica@umich.edu (E.C.J.); 3 Department of Biostatistics, University of Michigan School of Public Health, Ann Arbor, MI 48109, USA; pxsong@umich.edu; 4 National Institute of Public Health, Cuernavaca 62100, Mexico; libniavib@gmail.com (L.A.T.-O.); mmtellez@insp.mx (M.M.T.-R.)

**Keywords:** metabolic health, cardiovascular health, epigenetics, biomarkers, precision health, midlife, women’s health

## Abstract

**Background/Objectives**: Metabolic syndrome (MetS) is a cluster of metabolic risk factors that markedly increase the risk of cardiovascular disease and type 2 diabetes. Its prevalence exceeds 50% among Mexican women. A growing number of studies suggest that DNA methylation, an epigenetic mechanism, is linked to MetS. Methylation risk scores (MRS) may be able to predict future disease risk, yet prospective studies with MRS and MetS are rare, especially in Hispanic populations. We aimed to (1) replicate previously reported locus-specific DNA methylation–MetS associations, (2) identify additional loci, and (3) evaluate an MRS for predicting MetS among a sample of midlife Mexican women. **Methods**: We analyzed 149 women from the Early Life Exposure in Mexico to Environmental Toxicants (ELEMENT) cohort with DNA methylation measured in blood leukocytes collected around 2008 and MetS assessed approximately ten years later. Logistic regression was applied to a priori loci, and then all loci on the Infinium EPICv1 array. MRS performance was evaluated by cross-validation. **Results**: Four loci—probe ID cg11024682 (*SREBF1*), cg06500161 (*ABCG1*), cg22891070 (*HIF3A*), and cg02650017 (*PHOSPHO1*)—showed consistent associations with MetS. For example, each 1-percentage-point increase in methylation at cg11024682 (*SREBF1*) was associated with higher odds of MetS in the fully adjusted model (OR = 1.30; 95% CI: 1.12–1.52). No additional loci reached Bonferroni significance, though marginal associations with MetS components were observed. The MRS derived from four replicated loci was associated with higher odds of MetS per 1-SD increase in the training set (OR = 3.55; 95% CI: 2.03–6.22). In the independent testing set, the MRS-only model improved prediction compared with the baseline covariate-only model (AUC = 0.652 vs. 0.398; ΔAUC = +0.254). **Conclusions**: This study provides prospective evidence that specific DNA methylation markers are associated with MetS risk in midlife Mexican women. These loci, now replicated in several cohorts, are promising biomarkers for early identification and prevention of MetS.

## 1. Introduction

Metabolic Syndrome (MetS) is a cluster of interrelated metabolic risk factors that significantly heighten the risk of developing cardiovascular disease (CVD) and type 2 diabetes mellitus (T2D). The increasing prevalence of MetS worldwide [1,2] has sparked a renewed interest in understanding the biological mechanisms underlying its pathogenesis [3]. Both genetic predisposition and environmental influences contribute to the development of MetS. Epigenetic research offers the potential to unravel the molecular pathways through which lifestyle behaviors, such as diet and physical activity, interact with an individual’s genetic background to affect health outcomes. DNA methylation is the mitotically heritable addition of a methyl group to the fifth carbon of cytosine bases by DNA methyltransferase enzymes [4]. Among the various epigenetic modifications, DNA methylation is widely studied due to its relative stability over time, offering a potential mechanism through which environmental factors can influence gene expression, thereby altering long-term disease risk [5]. Moreover, DNA methylation measured in accessible tissues such as blood may serve as predictive biomarkers of disease that are useful for precision health or research purposes [6]. Aggregating information across multiple CpG loci into methylation risk scores (MRS)—the weighted sum of methylation levels across loci—has emerged as an increasingly utilized approach for predicting disease risk [7], with applications demonstrated in cancer and other chronic diseases [8,9].

Recent epigenome-wide association studies (EWAS) have explored the relationship between DNA methylation and MetS. These studies have identified significant associations between DNA methylation levels at specific CpG loci and MetS including in *CPT1A*, *ABCG1*, and *IGF2BP1* [3,5,10,11], further demonstrating the potential utility of MRS in predicting MetS. However, the majority of these studies employ cross-sectional designs to investigate the association between DNA methylation and MetS, limiting causal inference. Furthermore, the populations studied were predominantly of European ancestry, with some studies including African American participants [11]. Most of these studies focused on adult populations, although some, such as the family cohort study by Ali et al. (2016) [10], included children. Gender-specific analyses remain uncommon; for instance, Yoon et al. (2017) [12] included only men, while most other studies did not stratify by gender.

The prevalence of MetS among Mexican adults has risen steadily over the past few decades, with an overall prevalence of 50.3% among the Mexican population [13]. Notably, among this population women exhibit a higher prevalence than men (55.6% vs. 38.2%) [14]. Advanced age further elevates the odds of MetS [15]. It is important to understand population-specific DNA methylation patterns that may be linked to increased disease risk. Investigating these epigenetic alterations in middle-aged Mexican women could provide critical insights into the underlying mechanisms of MetS while also clarifying the utility of MRS for predicting MetS in this high-risk population, thereby informing targeted screening and prevention strategies.

No prospective study has evaluated whether DNA methylation markers or MRS predict later MetS specifically among Mexican or broader Hispanic midlife women, despite high MetS burden in this population and potential vulnerability during midlife metabolic and hormonal transitions. We conducted this study using data from midlife women recruited in Mexico City, an understudied population at risk for MetS, with the following objectives. First, we evaluated whether blood leukocyte DNA methylation at loci previously associated with MetS in multiple study populations replicate prospectively in this study population. Second, we conducted a pilot EWAS to identify additional loci associated with MetS. Third, we generated an MRS and tested its ability to predict MetS in midlife women approximately 10 years later. Together, this study addresses an important gap by evaluating whether previously reported MetS-related methylation markers generalize to an understudied population of Mexican women in a prospective setting, and by testing whether a small set of replicated CpG loci can provide predictive information for later-life MetS risk.

## 2. Results

### 2.1. Study Population Characteristics

Table 1 displays the demographic characteristics of the study sample, comprising 149 participants, of whom 87 have MetS at the second study visit. The average baseline age for participants with and without MetS at follow-up were 34.9 and 35.7 years, respectively. At the baseline, participants who subsequently had MetS had significantly higher BMI, waist circumference, and systolic and diastolic blood pressure than participants without MetS at follow-up. Among the estimated blood-cell proportions, only B-cell proportion differed significantly between the two groups. Socioeconomic status, assessed using the AMAI classification, did not differ significantly by follow-up MetS status.

Table 2 presents clinical characteristics, smoking status, and medication use at the follow-up visit. Significant differences between the two groups are observed in the clinical measures, including fasting glucose, triglycerides, systolic and diastolic blood pressure, and waist circumference. HDL cholesterol was lower among participants with MetS, although the difference did not reach statistical significance. Smoking status did not differ significantly by MetS status. Participants with MetS were more likely to report use of glucose-lowering, cholesterol-lowering, and triglyceride-lowering medications, whereas antihypertensive medication use did not differ significantly between groups.

As shown in Table S1, BMI, systolic and diastolic blood pressure, and waist circumference increased significantly from baseline to follow-up in both MetS groups. Participants with MetS at follow-up had higher values at both visits and showed larger mean increases in BMI, systolic blood pressure, and waist circumference than participants without MetS, whereas the mean increase in diastolic blood pressure was similar between groups.

### 2.2. Regression Results—A Priori Loci Analysis

DNA methylation CpG loci associated with MetS are presented in Figure 1 and Supplemental Table S2. All reported odds ratios (ORs) for MetS correspond to a 1-percentage increase in methylation (the locus β value). The lipogenesis-associated CpG locus (probe ID cg11024682), located in *SREBF1*, shows a consistent positive association with MetS across all models with ORs of 1.32 (95% CI: 1.15–1.52) in Model 1, 1.33 (95% CI: 1.14–1.54) in Model 2, 1.33 (95% CI: 1.14–1.55) in Model 3, and 1.29 (95% CI: 1.11–1.51) in Model 4. The phospholipid metabolism-associated locus (cg02650017), in the *PHOSPHO1* gene, shows a significant inverse association with MetS in Models 1–3, resulting in an OR of 0.78 (95% CI: 0.62–0.97) in Model 3; the association was attenuated after additional adjustment for baseline waist circumference in Model 4 (OR = 0.81, 95% CI: 0.64–1.02). The adiposity-related gene *HIF3A* (cg22891070) is positively associated with MetS across all models, with an OR of 1.04 (95% CI: 1.01–1.07) in each model. Lastly, a locus (cg06500161) located in the lipoprotein-related gene *ABCG1* shows a positive association with MetS in Model 2 and Model 3, with an OR of 1.16 (95% CI: 1.00–1.34) in Model 3; this association was attenuated and was no longer statistically significant after adjustment for baseline waist circumference in Model 4 (OR = 1.11, 95% CI: 0.95–1.30).

Model comparison results for the additional adjustment for baseline waist circumference are presented in Supplemental Table S3. The largest percentage change in the CpG regression coefficient between Models 3 and 4 was observed for *IGF2BP1* cg06638433 (87.0%); however, the coefficient was small in magnitude in both models (β = 0.008 in Model 3 and β = 0.001 in Model 4). For the remaining CpG loci, the percentage change in the regression coefficient ranged from 1.5% to 27.6%, except for *SOCS3* cg18181703, for which the coefficient increased in magnitude after adjustment for baseline waist circumference (% change = −12.7%). AIC values were lower in Model 4 than in Model 3 for all CpG-specific models, with ΔAIC values ranging from −5.82 to −9.98. Likelihood ratio tests further indicated improved model fit after inclusion of baseline waist circumference for all CpG-specific models (all *p* ≤ 0.005).

### 2.3. Epigenome-Wide Analysis

At the threshold of *p*-value < 1.1 × 10^−7^, no statistically significant associations were observed between DNA methylation of any CpG locus and MetS in the EWAS. The Manhattan plot in Figure 2 presents the association results from Model 2, examining the relationship between DNA methylation levels at CpG loci across the genome and binary MetS status. Using a marginal significance level of *p*-value < 1.0 × 10^−4^, 33 out of 770,085 CpG loci showed associations with binary MetS status (Table S4). Similarly, no statistically significant associations were found between methylated CpG loci and individual MetS components (as continuous variables) in the EWAS. However, in the analysis treating each MetS component as a binary outcome, using a marginal significance level of 1.0 × 10^−4^, there were associations with elevated triglycerides (11 CpG loci), elevated blood pressure (30 CpG loci), lower HDL cholesterol levels (6 CpG loci) and elevated fasting glucose (7 CpG loci) (Supplemental Tables S5–S8.)

### 2.4. Predictive Analysis Results—Selected Loci Analysis

Based on the model coefficients from Model 2 for 4 selected CpG loci (cg11024682 in *SREBF1*, cg02650017 in *PHOSPHO1*, cg22891070 in *HIF3A*, and cg06500161 in *ABCG1*) that were significantly associated with MetS in at least one model within the a priori analysis, the MRS was calculated. A two-sample *t*-test revealed a highly significant difference in standardized MRS between individuals with and without MetS ($p=3.25\times 10^{-8}$). Each one standard deviation increase in the MRS was associated with a 3.55-fold higher odds of having MetS (OR = 3.55; 95% CI: 2.03–6.22) in the MRS-only model (PModel 2). This association remained significant after adjustment for baseline covariates in PModel 3 (OR = 3.82, 95% CI: 2.11–6.93), further adjustment for estimated blood cell proportions in PModel 4 (OR = 4.74, 95% CI: 2.30–9.76), and additional adjustment for baseline waist circumference in PModel 5 (OR = 4.41, 95% CI: 2.09–9.34). All associations had a *p*-value < 0.001 (Supplemental Table S9).

The performance of the five prediction models based on five-fold cross-validation is summarized in Table 3. Incorporating the MRS improved MetS prediction, as evidenced by the higher cross-validated AUCs for PModels 2–5 compared with PModel 1. Among the models, PModel 2, which included only the standardized MRS, showed the best overall performance, with the highest mean AUC of 0.795 and accuracy of 0.695, compared with 0.497 and 0.571, respectively, for the covariate-only model. PModel 2 also maintained relatively high sensitivity (0.796) and substantially higher specificity (0.550) than PModel 1 (0.172). Although PModels 3–5 also outperformed the covariate-only model in terms of AUC, none exceeded the performance of the MRS-only model.

In the independent testing set, PModel 2 continued to show the best overall predictive performance, achieving the highest AUC (0.652), accuracy (0.614), specificity (0.650), precision (0.667), and NPV (0.565), together with the lowest Brier score (0.236) and log loss (0.661) (Table 4, Figure 3), indicating strong and balanced predictive performance. This model demonstrated robust performance, with the lowest trade-offs between sensitivity and specificity, and lowest prediction error, making it the preferred model for predicting MetS in this cohort. Furthermore, adding the MRS to baseline covariates (PModel 3) or further adjusting for immune cell composition (PModel 4) also improved performance relative to PModel 1, though to a lesser extent than PModel 2. PModel 5 achieved an AUC of 0.644 after additional adjustment for baseline waist circumference, which remained slightly lower than that of PModel 2. Overall, the independent testing results were consistent with the cross-validation findings and supported PModel 2 as the preferred model for MetS prediction in this cohort.

## 3. Discussion

Within this study of midlife Mexican women, we aimed to replicate previously reported associations between blood leukocyte DNA methylation at specific CpG loci and MetS with a prospective study design. While only two were statistically significant in all three models, the direction of these associations (7 out of 8) aligns with findings from previous studies (Table 5), reinforcing the robustness of the selected loci. This consistency across independent cohorts underscores the reliability of the identified DNA methylation markers in metabolic disease research across various populations.

Although this study did not identify novel CpG loci that reached epigenome-wide significance, it provides new evidence in three important ways. First, it demonstrates that several MetS-associated DNA methylation markers previously identified in other populations are prospectively associated with MetS approximately 10 years later in Mexican women. Second, it extends the evidence base for MetS-related methylation biomarkers to a high-risk and understudied Hispanic population. Third, it shows that a parsimonious MRS based on replicated loci has predictive value for later MetS, supporting the potential utility of these loci as early biomarkers rather than merely cross-sectional correlates of metabolic dysfunction.

Differences in the strength and statistical significance of associations observed in this and past studies may be attributable, in part, to differences in the tissue sources of DNA used across studies. While most prior EWAS of MetS have relied on whole blood or buffy coat–derived DNA [5,11], others have examined DNA methylation in isolated CD4+ T cells [3] or buccal epithelial cells [10]. Given well-established tissue-specific and cell-type-specific DNA methylation patterns, such differences in biological source may contribute to inconsistencies in DNA methylation–MetS associations observed across populations along with study design differences.

DNA methylation in the present study was measured in mixed blood leukocyte samples; variation in leukocyte composition may influence observed methylation profiles. We observed a modest but statistically significant difference in estimated B cell proportions between women with and without MetS, which may reflect immune dysregulation related to chronic low-grade inflammation in MetS. At the same time, differences in B cell proportions could contribute to apparent methylation differences in bulk blood. To address this, we adjusted for estimated immune cell proportions in the a priori logistic regression sensitivity analysis (Model 3) and in the MRS prediction sensitivity model (PModel 4). The replicated associations generally remained consistent after cell-composition adjustment, suggesting that the primary DNA methylation findings were not solely explained by differences in blood cell composition. However, because cell proportions were estimated and methylation was measured in bulk leukocytes, we cannot determine whether B cell-related immune shifts are a driver, mediator, or consequence of MetS-associated methylation profiles. Future cell-type-specific methylation studies are needed to clarify this relationship.

Among the a priori loci, four—cg11024682 in *SREBF1*, cg06500161 in *ABCG1*, cg22891070 in *HIF3A*, and cg02650017 in *PHOSPHO1*—demonstrated consistency across all three adjusted models and showed statistically significant associations with MetS in at least one model. This replication reinforces their potential to serve as biomarkers of MetS.

DNA methylation at locus cg11024682 in *SREBF1*, was found to be positively significantly associated with MetS in a Finnish population [16]. *SREBF1* encodes a transcription factor that plays a central role in regulating lipid homeostasis by activating genes involved in fatty acid and cholesterol biosynthesis [19]. Consistent with its metabolic functions, the DNA methylation level of cg11024682 in *SREBF1* has also been linked to liver fat accumulation [20], type 2 diabetes [21], and BMI [22], indicating its broader relevance to metabolic health.

Similarly, the *ABCG1* locus cg06500161 showed a positive association with MetS in this study, aligning with prior findings in an African American population [5] and a Finnish population [16]. *ABCG1* encodes a membrane transporter involved in cholesterol and phospholipid efflux, particularly facilitating cholesterol transfer from macrophages to HDL particles [23]. Methylation at this locus has also been linked to coronary artery disease [24]. Its consistent detection across several study populations reinforces its candidacy as a robust methylation marker for metabolic and CVD risk.

The *HIF3A* locus cg22891070, implicated in adiposity, has previously been linked to BMI and insulin sensitivity in European populations [18,25]. The current study confirms its prospective association with MetS in a Hispanic population, suggesting shared epigenetic pathways that transcend ancestry, in a gene regulating energy balance and adipogenesis. *HIF3A* encodes a member of the hypoxia-inducible factor (HIF) transcription factor family, which plays a role in cellular responses to low oxygen levels [26] and has been increasingly studied in relation to obesity and metabolic traits [27].

In contrast, higher methylation of cg02650017 in *PHOSPHO1* displayed variable directionality across studies, with negative associations in an African American cohort and positive associations in a White population [3]. This study reported a significant inverse association with MetS in Hispanic women which aligns with results from a recent multi-ethnic cohort [17], highlighting the potential for context-specific epigenetic effects influenced by population background or environmental exposures. *PHOSPHO1* encodes a phosphatase critical for bone mineralization [28]. Interestingly, mice deficient in the PHOSPHO1 enzyme exhibit improved glucose homeostasis and are resistant to diet-induced obesity and diabetes, suggesting a possible link between skeletal biology and systemic metabolic regulation [29]. These findings suggest that *PHOSPHO1* is a bone-derived regulator relevant to metabolic disease.

The *SOCS3* locus cg18181703 was positively associated (but not statistically significant) with MetS in our study, whereas Ali et al. reported an inverse association between *SOCS3* methylation and MetS. This discrepancy may reflect differences in study population, age distribution, biological sample type, MetS definition, timing of methylation measurement relative to disease status, or statistical analysis approaches. Ali et al. examined peripheral blood mononuclear cell DNA methylation in a family-based obesity study, whereas the current study used mixed blood leukocyte DNA methylation in midlife Mexican women with prospective MetS assessment approximately 10 years later. In addition, *SOCS3* is involved in leptin and insulin signaling, suggesting that its methylation may be related to metabolic regulation in a context-dependent manner.

Taken together, the replicated CpG loci may reflect interconnected pathways involved in metabolic dysregulation rather than isolated biomarkers. *SREBF1* and *ABCG1* are both closely related to lipid metabolism, with *SREBF1* involved in lipogenesis and cholesterol biosynthesis and *ABCG1* involved in cholesterol and phospholipid efflux. Altered methylation at loci within these genes may therefore influence dysregulation of lipid storage, transport, and circulating lipid profiles that are central to MetS. *HIF3A* may link adiposity-related methylation changes to hypoxia signaling, which is relevant because adipose tissue expansion can promote local hypoxia, inflammation, and insulin resistance. *PHOSPHO1* may represent an additional connection between phospholipid metabolism and systemic metabolic regulation. Together, these loci point toward a broader biological model in which altered lipid handling, adipose hypoxia, immune-inflammatory signaling, and metabolic aging contribute to MetS development. However, because this study was observational and based on blood leukocyte DNA methylation, these mechanisms should be interpreted as biologically plausible hypotheses requiring functional validation.

Since the study population and design was unique compared to past studies (Hispanic population, women-only, and prospective design), we anticipated additional loci to be associated with MetS that were not previously identified. Thus, we conducted an EWAS of MetS in this pilot sample of middle-aged women with heightened susceptibility to MetS. While there were no statistically significant findings after accounting for multiple comparisons, the small sample size limited statistical power in this analysis. Using a marginal significance threshold (1.0 × 10^−4^), we identified 33 CpG loci associated with MetS, and an additional 11, 30, 6, and 7 CpG loci associated with elevated triglycerides, elevated blood pressure, low HDL cholesterol, and elevated fasting glucose, respectively. Based on the EWAS Atlas open platform [30], information about traits these loci have previously been linked to are shown in Supplemental Tables S4–S8.

One locus was identified as marginally significant in both the MetS and triglyceride analysis—cg05997357 in *NCOA2*. *NCOA2* encodes a nuclear receptor coactivator involved in a range of endocrine and metabolic functions and has been implicated in glucose homeostasis, lipid regulation, and tumorigenesis [31]. Among the top loci associated with MetS in the EWAS, cg08242636 in *CBFB* was previously reported to have higher DNA methylation levels in individuals with T2D compared to non-diabetic controls [32]. *CBFB* encodes the core-binding factor beta (CBFβ) subunit, which forms a heterodimeric complex with RUNX proteins to regulate the transcription of genes involved in hematopoiesis [33]. Although primarily studied in the context of blood cell development, emerging evidence suggests that CBFβ may also play a broader role in metabolic regulation through transcriptional control mechanisms.

Another noteworthy finding is cg04348086 in *ATP2B2*, associated with elevated blood pressure analysis and previously linked to T2D [32]. While *ATP2B2* is traditionally studied in the context of calcium signaling in excitable tissues, its emerging metabolic role reflects the broader involvement of ion transport pathways in vascular and metabolic homeostasis. Finally, 17 CpG loci emerging from the EWAS were annotated to genes related to the aging process. This observation aligns with prior evidence that epigenetic aging and metabolic dysfunction share overlapping biological pathways [34,35]; it may also point to a deeper interplay between the aging DNA methylome and MetS risk. Other marginally significant loci were annotated to traits such as smoking, air pollution, aging, and gestational diabetes, suggesting potential links to environmental exposures and metabolic aging. These findings should be interpreted as hypothesis-generating and require replication.

The predictive analysis provides further evidence that DNA methylation can be used for disease prediction. Using four CpG loci significantly associated with MetS in this study population and several others, we developed an MRS that performs well in middle-aged Hispanic women, with more than 10 years between DNA methylation measurement and MetS assessment. This temporal gap strengthens the evidence for the predictive value of epigenetic markers and supports the potential role of MRS as a clinically useful early biomarker for MetS.

The findings reported in this study are consistent with those of the only prior study to date applying an MRS for MetS prediction [11]. In that study, an MRS was constructed using six CpG loci identified from EWAS of MetS and its individual components. The authors reported that each standard deviation (SD) increase in the MRS was associated with a 2.25- to 2.45-fold increase in the odds of MetS across two large cohorts (HyperGEN and GOLDN), with the strongest associations observed for waist circumference, triglycerides, and glucose levels. In the current study, we observed a similar effect size (OR = 2.51). While previous studies primarily involved U.S. cohorts of European or African ancestry, this study focuses on a Hispanic cohort, suggesting broader applicability of the MRS approach across populations.

Furthermore, model performance evaluation demonstrated that incorporating the MRS substantially improved MetS prediction. PModel 2—based solely on MRS—achieved higher accuracy, sensitivity, specificity, and a lower prediction MSE, underscoring the independent predictive utility of the MRS. Although the baseline covariates included in PModel 1 are known risk factors and showed strong associations with MetS in the prior literature, they did not provide high predictive accuracy in the study sample.

Beyond MetS itself, a growing number of studies have applied MRS approaches to predict individual MetS components or related outcomes. For example, Braun et al. (2017) [36], using a population-based cohort in Rotterdam, found that MRS constructed from EWAS signals was inversely associated with HDL cholesterol and positively associated with triglycerides. Other studies have extended this framework to predict future risk of T2D. A large transethnic cohort [37] showed that an MRS was strongly associated with incident T2D (relative risk = 1.41 per 1 SD increase in the score, *p* = 1.3 × 10^−26^). More recently, studies in Asian and multi-ethnic populations have further supported the applicability of MRS for predicting metabolic outcomes. In a Chinese cohort, [38] constructed an MRS from ten CpG sites and found that each SD increase in the score was associated with 1.68 higher odds of T2D, even after adjusting for behavioral and sociodemographic factors. Similarly, Linares-Pineda et al., 2024 [39] applied an MRS derived from a T2D EWAS meta-analysis to a diverse pregnancy cohort, STORK-G, and found significant associations between the MRS and fasting glucose levels across women of European, South Asian, African, Middle Eastern, and South American backgrounds. These findings lend support to the potential of MRS as tools for predicting metabolic outcomes in multi-ethnic populations.

Findings suggest that MRS could serve as a robust biomarker for evaluating MetS risk, especially in populations with limited representation in prior studies. Further replication in external cohorts and diverse groups will be crucial for validating the generalizability of the MRS presented here. Although approximately 45% of participants exceeded the waist circumference threshold for central adiposity at baseline, this proportion increased to 96% at follow-up, indicating a substantial progression of metabolic risk in the cohort. Accordingly, the MRS may be most applicable to populations with similar baseline risk profiles, and its performance may differ in younger, leaner, or lower-risk populations with greater variability in adiposity. Given that this signature is observed years before disease, it could be used as a precision health tool to identify individuals most in need of monitoring and prevention/intervention strategies. The inclusion of only 4 CpG loci means DNA methylation could be quantified using high throughput and cost-effective target-locus methods.

This study had many strengths including its prospective design, which allows for the temporal assessment of DNA methylation and subsequent MetS development. Furthermore, the replication of findings in an understudied population, specifically Mexican women, addresses a significant gap in the literature and enhances the applicability of the results.

However, the study has several limitations. First, the relatively small sample size may have limited statistical power for both the a priori analysis and the EWAS, particularly for detecting modest methylation–MetS associations or identifying novel epigenetic signals after correction for multiple testing. The retrospective power analysis provides a quantitative context for this limitation: under the specified assumptions and a 5% FDR threshold, the sample size provided an estimated 80% power to detect a between-group methylation difference of 3.3 percent. Smaller differences may therefore have gone undetected. This estimate is conditional on the assumed proportion of truly associated loci and the FDR criterion; the primary EWAS used a more stringent Bonferroni threshold. Larger studies will be needed to detect smaller effects with confidence. Second, a primary limitation is the lack of an external validation cohort. Although we evaluated the MRS using internal training and testing procedures, the predictive performance and generalizability of the MRS have not been tested in an independent cohort. Third, DNA methylation was measured in blood leukocytes, a mixed immune-cell population. Although we adjusted for estimated leukocyte proportions in sensitivity analyses, blood leukocyte methylation may partly reflect immune cell composition and may not capture methylation patterns in target metabolic tissues such as liver, adipose tissue, or skeletal muscle. Fourth, the study enrolled women only and may not be generalizable to men. In addition, participants were drawn from a single urban cohort in Mexico City, which may limit the representativeness of the findings for rural Mexican women or Hispanic women in other geographic contexts. Fifth, we did not have complete information on medication use for triglycerides, HDL cholesterol, glucose, or hypertension at the baseline study visit. As a result, we could not evaluate whether pharmacologic treatment before or at the time of DNA methylation assessment may have influenced methylation profiles. Complete interim clinical assessments of all MetS components were also not available between the baseline DNA methylation visit and the midlife follow-up visit. Therefore, although DNA methylation was measured before follow-up MetS classification, the exact timing of MetS onset is unknown. These limitations may introduce potential bias, limit our ability to characterize disease progression during the intervening period, and underscore the need for future studies with repeated clinical assessments and medication histories. Finally, DNA methylation was measured using the Illumina EPIC array rather than whole-genome bisulfite sequencing. Therefore, our analysis was restricted to CpG loci represented on the array. However, the EPIC array provides broad coverage of regulatory and disease-relevant loci and enables comparison with prior EWAS of MetS that used array-based methylation profiling. Future studies using whole-genome bisulfite sequencing or other sequencing-based approaches in larger, more diverse, and externally validated prospective cohorts may identify additional loci and provide a more comprehensive characterization of the DNA methylome in relation to MetS.

In conclusion, this study supports the role of DNA methylation at specific loci as promising candidate biomarkers of MetS, particularly in middle-aged Hispanic women and provides potential insight into genes important in the pathogenesis of MetS. The replicated loci—in the genes *SREBF1*, *ABCG1*, *HIF3A* and *PHOSPHO1*—underscore the involvement of metabolic and inflammatory pathways in MetS. Altogether, these findings support the potential utility of epigenetic markers as indicators of long-term metabolic risk. Importantly, their consistent effects in our prospective study design strengthen their utility as prospective biomarkers. Replication and external validation in larger, independent, multi-ethnic cohorts are needed before these markers can be considered for precision medicine or clinical risk prediction. Targeted epigenetic research in diverse populations remains critical for developing translatable prevention strategies. Further research with larger sample sizes, external validation cohorts, and more precise temporal data on disease onset is needed to fully elucidate the epigenetic mechanisms underlying MetS and to identify potential biomarkers for early detection and intervention.

## 4. Materials and Methods

### 4.1. Study Population

Participants from the Early Life Exposure in Mexico to Environmental Toxicants (ELEMENT) Project were included in this research. Briefly, the ELEMENT study was established to investigate the long-term consequences of toxicant exposures during the perinatal period on children’s health. A total of 1643 mother–child pairs were recruited between 1994 and 2003 during pregnancy or at delivery from maternity hospitals in Mexico City, as described previously [40]. Mothers and their children were followed up periodically and monitored for various chemical exposures, lifestyle factors such as diet, and health outcomes ever since. The current study includes data from a follow-up visit that occurred around 2008 and another study visit focused on women’s midlife health that started in 2019. We selected a subset of women who had available DNA methylation data from the 2008 follow-up visit and who were also followed-up at the second study visit (about 10 years later) for MetS assessment (n = 149 participants).

### 4.2. Metabolic Syndrome

The components of MetS were assessed during the study visit that started in 2019. Trained research staff collected anthropometric and physiological measurements, including weight, height, and waist circumference, with body mass index (BMI) calculated as kg/m^2^. Waist circumference was measured to the nearest 0.1 cm. Fasting venous blood samples were drawn, and serum was separated for enzymatic analysis of glucose, triglycerides, total cholesterol, and high-density lipoprotein (HDL) cholesterol, with corrections for endogenous glycerol at the Institute of Perinatology (INPer) in Mexico. Blood pressure, both diastolic and systolic, was measured by trained personnel using a standardized protocol and a Spacelabs Healthcare automated oscillometric device. To classify presence of MetS, the criteria established by the National Cholesterol Education Program (NCEP) Adult Treatment Panel III (ATP III) were used, which was subsequently updated by the American Heart Association and the National Heart, Lung, and Blood Institute in 2005 and applied in previous ELEMENT work [41]. Briefly, the NCEPT ATP III criteria provide cutoffs to determine elevated waist circumference, hypertension, hypercholesterolemia, hypertriglyceridemia, and hyperglycemia. Meeting the criteria for at least 3 out of 5 parameters is defined as MetS. In an effort to accurately determine MetS status, four types of medication were considered. These included medication for treating high triglycerides, low HDL cholesterol, high glucose levels, and hypertension. If a participant reported using any of these medications, they were categorized accordingly—for example, those taking medication to reduce high triglycerides were considered as meeting the triglyceride criterion as well. Based on these criteria, we constructed a binomial variable to categorize individuals as either having MetS or not.

### 4.3. Covariates

Covariates were selected based on a priori knowledge. To enhance the precision of socioeconomic status (SES) measurement, a classification by the Asociación Mexicana de Agencias de Investigación de Mercado y Opinión Pública (AMAI) was used which evaluates household resources to group individuals into seven categories ranging from A/B (highest) to E (lowest) [42]. Due to small sample sizes in the highest and lowest SES tiers, the A/B category was merged into C+ and similarly the E category was combined into D. All covariates—including SES, age and smoking status—were assessed at baseline. Smoking status was categorized as never, former, or current smoker.

### 4.4. DNA Methylation Analysis

The Illumina Infinium MethylationEPIC array (‘EPIC’; Illumina, Inc.; San Diego, CA, USA) was used to assess DNA methylation with DNA extracted from blood leukocytes collected at the 2008 study visit. DNA was isolated via Puregene kits (Gentra Systems; Minneapolis, MN, USA) within a few years of blood collection and stored frozen at −80 °C until DNA methylation analysis. The EPIC array quantifies DNA methylation at over 850,000 cytosine–phosphate–guanine (CpG) loci. Briefly, DNA quantity and quality were measured via a QuantiFluor dsDNA System (Promega; Madison, WI, USA) or a Qubit Fluorometer (Thermo Scientific; Waltham, MA, USA) prior to bisulfite conversion of approximately 750 ng per sample with EZ DNA kits (Zymo Research, Irvine, CA, USA). Bisulfite converted DNA was randomized across chips and chip positions prior to standard Illumina amplification, hybridization, and imaging steps [43] conducted at the University of Michigan Advanced Genomics Core. For data processing, raw image files were read with the R package minfi [44], and quality control and normalization occurred using the package ENmix version 1.48 [45]. Probes were removed if at least 5% of samples were not detected (*p*-value > 1 × 10^−16^ compared to background). Background correction was performed with noob and dye bias correction with RELIC followed by quantile normalization [46]. Probes that are known to be cross-reactive (using the lists from McCartney et al., 2016 [47] and Pidsley et al., 2016 [46]) or have single nucleotide polymorphisms in the CpG or single-base extension site or Y chromosome probes were removed. Three samples failed quality control procedures and were excluded from analysis. Two samples were replicated for quality control purposes, and one replicate per person was included in final analyses. Beta (β) scores (i.e., the proportion of methylation ranging from 0 to 1) of the processed data were used as final outcomes in the analysis. For cell composition, the established algorithm [48] was applied to estimate the proportions of various immune cells including CD4+ T cells, CD8+ T cells, natural killer cells, B cells, monocytes, and granulocytes [49].

The loci for the a priori analysis (Table 6) for this study were selected based on their associations with MetS in at least two different adult cohorts, as identified in previous EWAS. These loci are located in genes involved in key metabolic processes, such as lipid metabolism and inflammation, which are crucial in the development of MetS. The consistent replication of these associations across diverse populations provided the rationale for inclusion in this analysis.

### 4.5. Statistical Methods

The proportion of participants meeting the MetS criteria was determined, and chi-square tests and *t*-tests were utilized to examine differences in sociodemographic variables by MetS status. Logistic regression models were employed with MetS as the outcome and individual CpG β scores (proportion methylation) as the predictors, adjusting for socioeconomic status (AMAI), smoking status, and chronological age at the time of DNA methylation analysis in Model 1. Model 2 further adjusted for the time interval between the two study visits when DNA methylation was measured (first visit) and when MetS was evaluated (second visit) since the time varied slightly between participants. A sensitivity analysis (Model 3) included additional adjustments for cell composition (CD8+ T cells, CD4+ T cells, natural killer cells, B cells, and monocytes). A sensitivity analysis (Model 4) further adjusted for baseline waist circumference to evaluate whether the associations between DNA methylation and MetS were independent of baseline central adiposity.

Model 1:

$$\mathrm{MetS}\;\sim \;\mathrm{AMAI}\;+\;\mathrm{smoking}\;+\;\mathrm{Chronological}\;\mathrm{Age}\;+\;{\mathrm{CpG}}_{\mathrm{beta}\;\mathrm{score}}$$

Model 2:

$$\mathrm{MetS}\;\sim \;\mathrm{AMAI}\;+\;\mathrm{smoking}\;+\;\mathrm{Chronological}\;\mathrm{Age}\;+{\;\mathrm{CpG}}_{\mathrm{beta}\;\mathrm{score}}\;+\;\mathrm{Time}\;\mathrm{Interval}$$

Model 3:

$$\mathrm{MetS}\;\sim \;\mathrm{AMAI}\;+\;\mathrm{smoking}\;+\;\mathrm{Chronological}\;\mathrm{Age}\;+{\;\mathrm{CpG}}_{\mathrm{beta}\;\mathrm{score}}\;+\;\mathrm{Time}\;\mathrm{Interval}\;+\;\mathrm{Cell}\;\mathrm{Type}$$

Model 4:

$$\begin{array}{c} \mathrm{MetS}\;\sim \;\mathrm{AMAI}\;+\mathrm{smoking}+\;\mathrm{Chronological}\;\mathrm{Age}\;+{\mathrm{CpG}}_{\mathrm{beta}\;\mathrm{score}}\;+\;\mathrm{Time}\;\mathrm{Interval}\;+\;\mathrm{Cell}\;\mathrm{Type}\\ +\;\mathrm{baseline}\;\mathrm{waist}\;\mathrm{circumference}\; \end{array}$$

Models were run first on the selected CpG loci (Table 6) that were robustly associated with MetS in previous studies for the a priori analysis. A *p*-value < 0.05 was considered statistically significant. To evaluate the impact of additionally adjusting for baseline waist circumference, Model 3 and Model 4 were compared for each CpG locus. The percent change in the CpG regression coefficient was calculated. Model fit was further compared using the Akaike information criterion (AIC) and likelihood ratio tests because Model 3 was nested within Model 4. The change in AIC was calculated as AIC_Model 4_ − AIC_Model 3_, with negative values indicating improved model fit after inclusion of the baseline waist circumference.

An EWAS was then performed to identify additional loci associated with MetS in this population. The binary variable for MetS status and binary indicators for the presence of individual MetS components were analyzed as outcomes in separate models for the EWAS, with the exception of waist circumference, which was excluded due to the limited variability in our study population—96% of participants had measurements exceeding 88 cm. In the EWAS, models were run for 770,085 CpG loci that passed quality control. To robustly control the family-wise error rate given the large number of tests, a Bonferroni correction was applied, setting the significance threshold at α = 0.05/770,000 ≈ 1.1 × 10^−7^ [50]. Results were visualized through Manhattan plots, illustrating the findings of the association analysis. Because no loci reached the Bonferroni-corrected threshold or FDR-based significance in the EWAS, loci with *p*-values < 1.0 × 10^−4^ are reported as exploratory, hypothesis-generating results.

To contextualize the absence of epigenome-wide significant findings and inform the design of future studies, a retrospective, simulation-based power analysis was conducted using the EpipwR package in R [51], which builds on the pwrEWAS framework for binary outcomes [52]. The analysis specified 86 participants with MetS and 62 without MetS, 770,085 loci, an assumed proportion of 1% truly associated loci, and a false discovery rate (FDR) threshold of 5%. Under these assumptions, the estimated power was 80% to detect a 3.3-percent difference in mean DNA methylation between groups. The margin of error for the simulated power estimate was less than 3 percentage points.

### 4.6. Calculation of MRS

To assess the predictive power of MRS in the context of MetS, several statistical approaches were employed. The following describes the calculation of MRS, model testing strategies, and evaluation methods applied in the analysis.

The MRS was constructed using the four replicated CpG loci, cg11024682 (*SREBF1*), cg02650017 (*PHOSPHO1*), cg22891070 (*HIF3A*), and cg06500161 (*ABCG1*), which were identified as significant in the adjusted model (Model 2) from the a priori analysis. The full analytic sample was first randomly partitioned into a training set (70%) and an independent testing set (30%) to prevent information from the testing set from contributing to MRS construction or model development (Figure 4).

Within the training data, CpG-specific weights were estimated from separate logistic regression models for MetS, adjusting for socioeconomic status, smoking status, chronological age, and the time interval between study visits (Model 2). The regression coefficients from the model for the association between a given locus and MetS were used as weights for each site. For participant *j*, the MRS was calculated as:

$${MRS}_{j}=\sum_{i=1}^{p}\beta_{i}\;\times \;X_{ij}$$

where $\beta_{i}$ represents the training-derived logistic regression coefficient for CpG *i*, $X_{ij}$ represents the methylation level of CpG *i* for participant *j*, and *p* = 4 represents the number of CpG loci included in the score.

The MRS was subsequently standardized using the mean and standard deviation estimated from the corresponding training data:

$${MRS}_{standardized}=\frac{MRS-\bar{{MRS}_{training}}}{SD({MRS}_{training})}$$

For internal cross-validation, CpG weights and MRS standardization parameters were re-estimated separately within each training fold and then applied to the corresponding held-out validation fold. For final evaluation in the independent testing set, CpG weights and standardization parameters were re-estimated using the complete 70% training set and then applied unchanged to the 30% testing set.

### 4.7. Predictive Models

Four logistic regression models were developed to evaluate the predictive contribution of the standardized MRS to MetS:

- PModel 1: Included only the covariates socioeconomic status, smoking status, chronological age, and the time interval between the two study visits. This baseline model served as a reference for prediction accuracy without the inclusion of MRS.
- PModel 2: Evaluated the standardized MRS as the sole predictor of MetS, assessing its independent predictive power.
- PModel 3: Combined the standardized MRS with covariates from PModel 1, assessing whether MRS improved prediction beyond standard covariates.
- PModel 4: Further included adjustments for cell composition to account for variability in immune cell proportions to account for variability in blood cell type proportions.
- PModel 5: Further included baseline waist circumference in addition to the standardized MRS, baseline covariates, and estimated immune cell composition to evaluate whether predictive performance remained independent of central adiposity.

### 4.8. Model Performance Evaluation

Model performance was evaluated using both five-fold cross-validation within the 70% training set and final evaluation in the independent 30% testing set (Figure 4). To minimize information leakage during cross-validation, MRS construction was incorporated within each cross-validation iteration. Specifically, for each fold, CpG-specific weights were estimated using only the four training folds. These weights were used to calculate the MRS in both the fold-specific training subset and the held-out validation fold. The mean and standard deviation used to standardize the MRS were likewise estimated exclusively from the fold-specific training subset and then applied to the validation fold. Each predictive model was subsequently fitted in the fold-specific training data and evaluated in the held-out fold. Cross-validation performance was summarized as the mean and standard deviation across the five validation folds.

Following cross-validation, the four CpG weights and MRS standardization parameters were re-estimated using the complete 70% training set. These training-derived parameters were then applied to the independent 30% testing set. The final versions of PModels 1–4 were fitted using the complete training set and evaluated in the testing set without further model fitting or parameter estimation [53].

Predictive performance was assessed using the area under the receiver operating characteristic curve (AUC), accuracy, sensitivity, specificity, precision, negative predictive value (NPV), F1 score, Brier score, and log loss. Accuracy, sensitivity, specificity, precision, NPV, and F1 score were calculated using a predicted probability threshold of 0.50 [54]. Receiver Operating Characteristic (ROC) curves were generated to visualize the trade-offs between sensitivity and specificity across classification thresholds. The AUC was calculated for each model to quantify the overall ability of the model to distinguish between participants with and without MetS. Sensitivity and specificity were calculated, providing an indication of each model’s performance in terms of true positive and true negative rates. The F1 score was calculated as the harmonic mean of precision and sensitivity, balancing false positives and false negatives. The Brier score and log loss were used to assess probabilistic prediction performance, with lower values indicating better prediction.

## Figures and Tables

**Figure 1:**
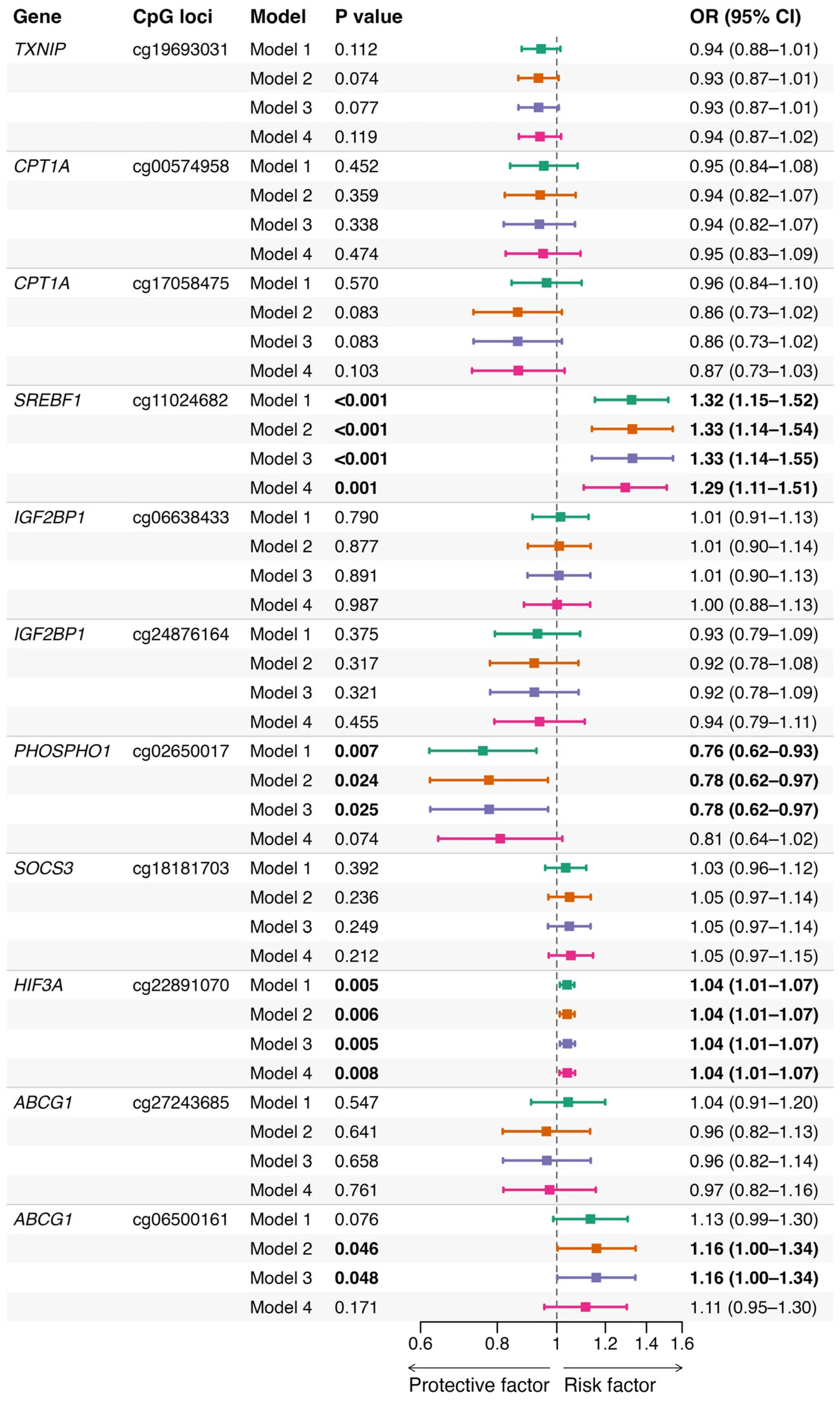
Forest Plot of Odds Ratios (ORs) for the association between DNA methylation and MetS at a priori CpG loci. Green, orange, purple and rose pink points with horizontal lines represent the OR and 95% confidence interval for Models 1, 2, 3 and 4, respectively. Dashed gray vertical line is for no effect (OR = 1.0). Points to the left suggest higher levels of methylation are protective, whereas points to the right indicate higher methylation at the locus is a risk factor for MetS. Bold text denotes associations with *p* < 0.05. ORs are reported per 1-percentage-point increase in DNA methylation.

**Figure 2:**
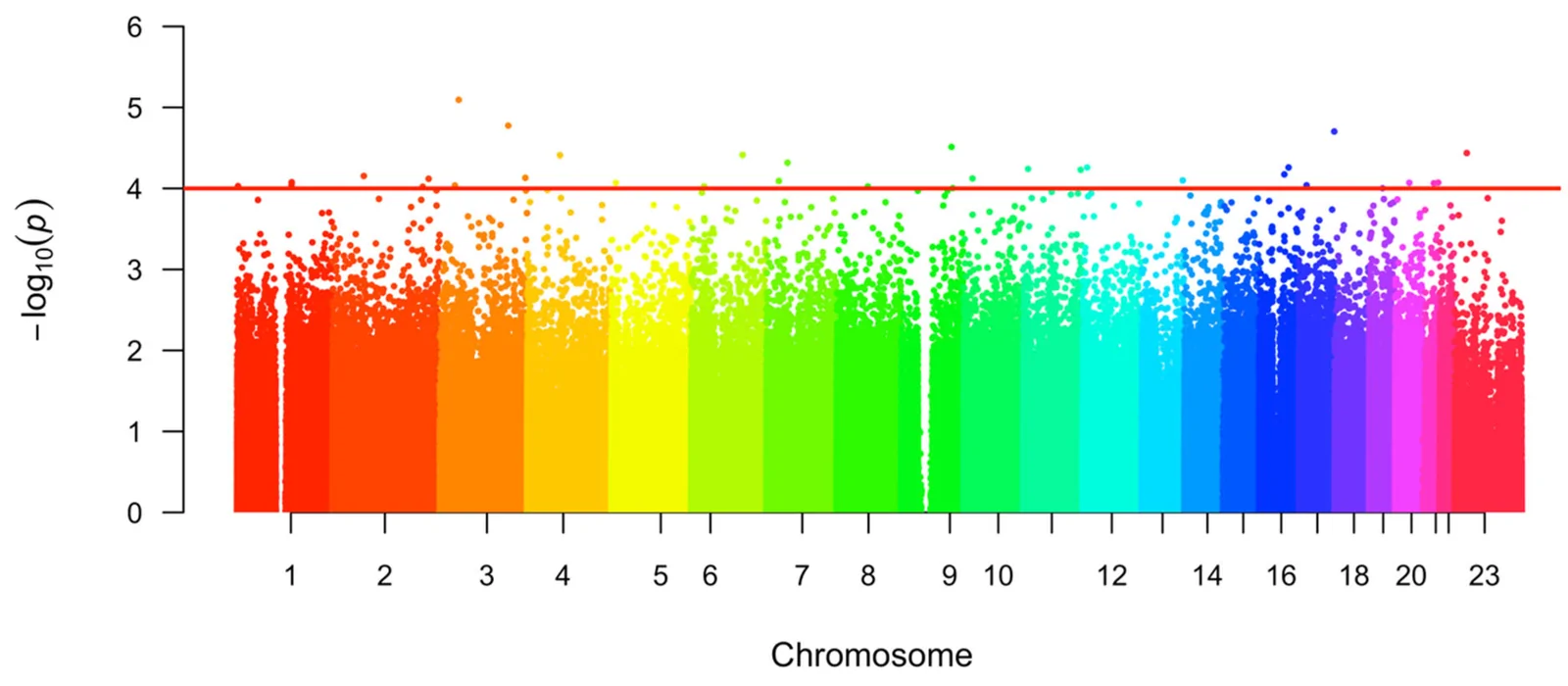
Manhattan plot for the epigenome-wide association study examining relationships between DNA methylation at all loci and MetS. MetS: metabolic syndrome. The red line indicates a marginal significance level of $1.0\;\times \;10^{-4}$. Dots represent results from individual loci, colored by chromosome. Models adjusted for SES, age, smoking status, and time between visits. No sites were significant at a Bonferroni correction cut-off.

**Figure 3:**
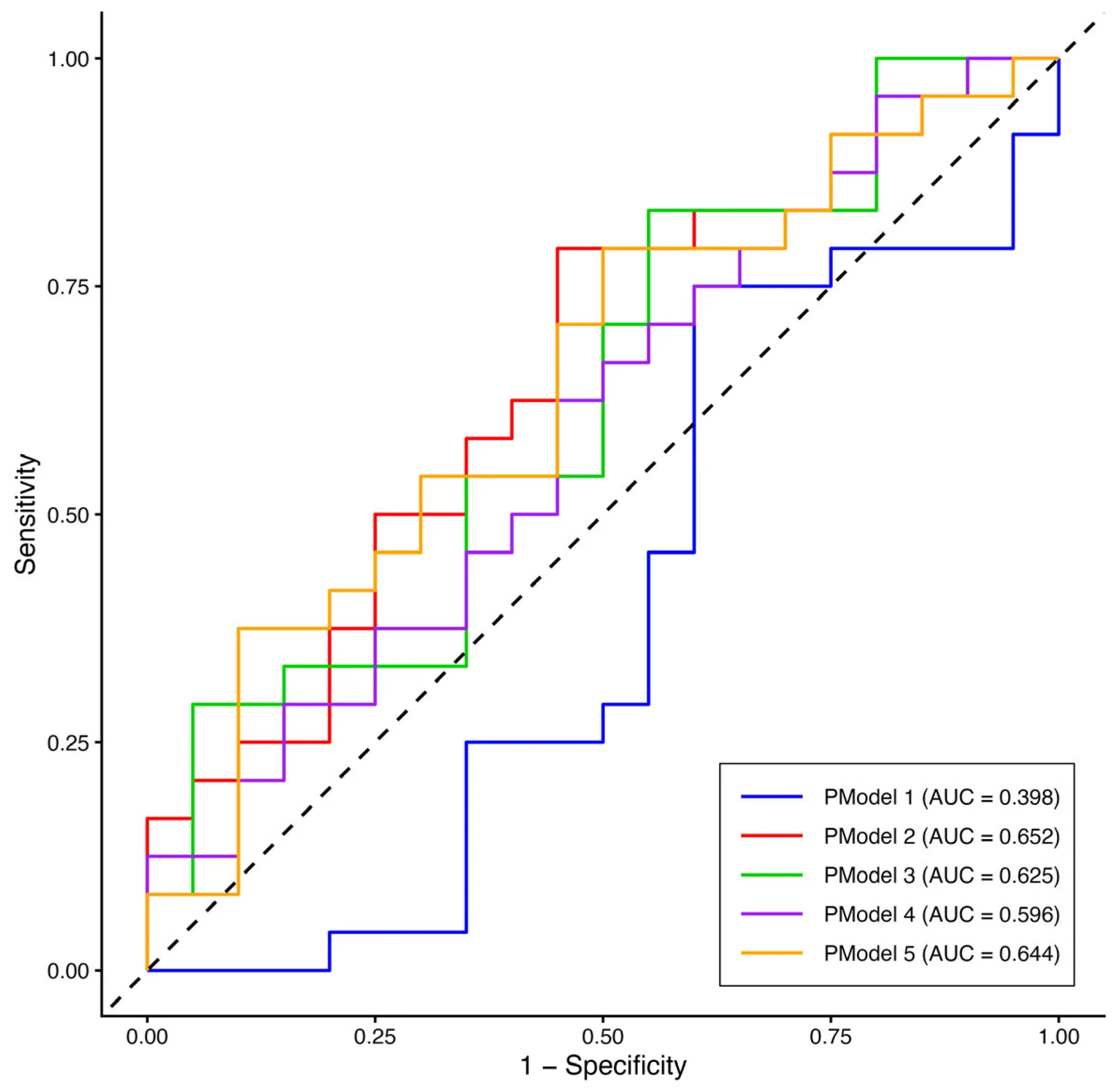
ROC Curves for All Models Predicting MetS (Independent Testing Set). The diagonal line represents a random classifier with an AUC of 0.5, and the curves above this line show improved model performance. PModel 1 (blue line) includes only covariates (SES, smoking status, chronological age, and time interval between visits). PModel 2 (red line) uses the MRS alone as the predictor. PModel 3 (green line) includes both the MRS and covariates. PModel 4 (purple line) further includes adjustments for estimated cell type proportions in addition to MRS and covariates. PModel 5 (orange line) further included baseline waist circumference in addition to MRS, baseline covariates, and estimated immune cell composition.

**Figure 4:**
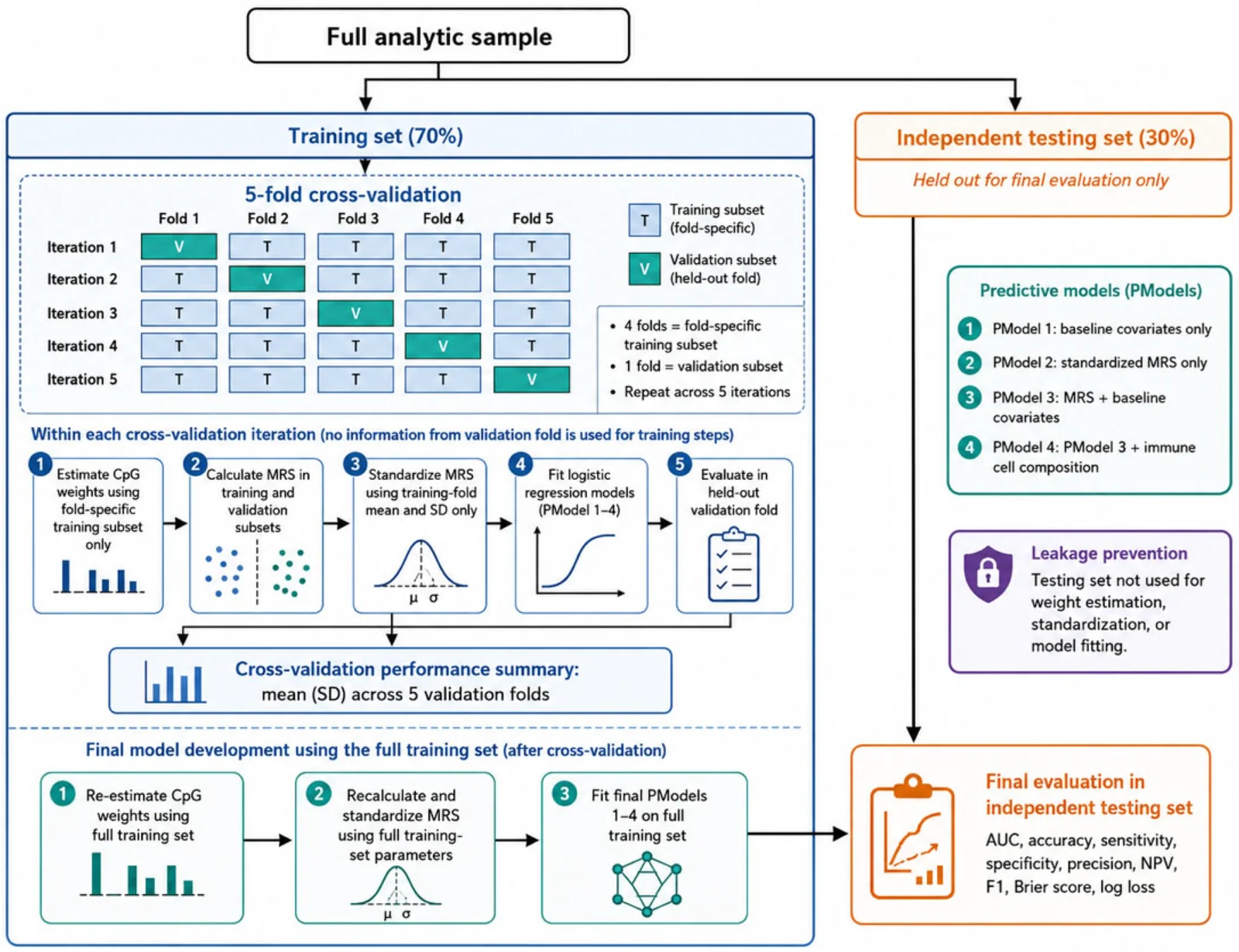
Predictive Modeling Workflow for MRS-Based MetS Prediction.

**Table 1:** Baseline characteristics stratified by metabolic syndrome status at the follow-up visit.

	Follow-Up MetS Status		
Baseline Characteristic	MetS− N = 62 ^1^	MetS+ N = 87 ^1^	*p* Value ^2^
Age (years)	34.9 (5.1)	35.7 (5.1)	0.360
BMI (kg/m^2^)	27.61 (4.57)	29.86 (4.84)	0.005
Waist circumference (cm)	84.23 (9.80)	89.37 (10.08)	0.002
Systolic blood pressure (mmHg)	103.19 (11.51)	110.05 (11.68)	<0.001
Diastolic blood pressure (mmHg)	68.05 (9.25)	72.36 (7.84)	0.003
Cell composition			
CD8+ T cells	0.05 (0.04)	0.06 (0.04)	0.219
CD4+ T cells	0.13 (0.05)	0.14 (0.05)	0.290
NK cells	0.03 (0.03)	0.04 (0.03)	0.334
B cells	0.06 (0.03)	0.07 (0.04)	0.041
Monocytes	0.05 (0.03)	0.05 (0.02)	0.474
Granulocytes	0.66 (0.09)	0.64 (0.09)	0.076
AMAI			0.997
A/B & C+	13 (21%)	19 (22%)	
C	21 (34%)	28 (32%)	
D+	18 (29%)	26 (30%)	
D & E	10 (16%)	14 (16%)	

^1^ Mean (SD); n (%). ^2^ Significance determined using Welch’s two-sample *t*-test for continuous variables; Pearson’s chi-squared test for categorical variables.

**Table 2:** Clinical characteristics, smoking status, and medication use stratified by metabolic syndrome status at the follow-up visit.

	Follow-Up MetS Status		
Follow-Up Characteristic	MetS− N = 62 ^1^	MetS+ N = 87 ^1^	*p* Value ^2^
Time interval (years)	9.97 (0.52)	10.00 (0.50)	0.734
BMI (kg/m^2^)	28.93 (4.36)	31.35 (4.89)	0.002
Waist circumference (cm)	97.66 (9.61)	104.17 (11.34)	<0.001
Systolic blood pressure (mmHg)	111.81 (12.84)	121.18 (18.65)	<0.001
Diastolic blood pressure (mmHg)	71.85 (7.32)	76.02 (10.21)	0.004
Fasting glucose (mg/dL)	87.72 (9.38)	101.51 (28.45)	<0.001
HDL cholesterol (mg/dL)	49.78 (10.09)	46.41 (10.54)	0.051
Triglycerides (mg/dL)	105.65 (30.18)	175.54 (104.17)	<0.001
Smoking status			0.799
Current	16 (26%)	22 (25%)	
Former	27 (44%)	34 (39%)	
Never	19 (31%)	31 (36%)	
Medication use			
Glucose-lowering medication	1 (2%)	21 (24%)	<0.001
Antihypertensive medication	3 (5%)	3 (3%)	0.693
Cholesterol-lowering medication	1 (2%)	9 (10%)	0.046
Triglyceride-lowering medication	1 (2%)	25 (29%)	<0.001

^1^ Mean (SD); n (%). ^2^ Significance was determined using Welch’s two-sample *t*-test for continuous variables, Pearson’s chi-squared test for smoking status, and Fisher’s exact test for medication-use variables.

**Table 3:** Five-Fold Cross-Validation Performance of Predictive Models.

	AUC	Accuracy	Sensitivity	Specificity
PModel 1	0.497 (0.119)	0.571 (0.034)	0.844 (0.122)	0.172 (0.144)
PModel 2	0.795 (0.096)	0.695 (0.080)	0.796 (0.100)	0.550 (0.119)
PModel 3	0.761 (0.093)	0.686 (0.093)	0.797 (0.149)	0.528 (0.150)
PModel 4	0.673 (0.112)	0.667 (0.101)	0.777 (0.040)	0.500 (0.215)
PModel 5	0.680 (0.084)	0.629 (0.071)	0.745 (0.045)	0.456 (0.159)

Values are presented as mean (standard deviation) across five validation folds. CpG weights and MRS standardization parameters were estimated separately within each training fold and applied to the corresponding held-out validation fold. PModel 1 includes baseline covariates: SES, smoking status, chronological age, and the time interval between study visits. PModel 2 includes only the standardized MRS to evaluate its independent predictive power. PModel 3 combines the MRS with all covariates from PModel 1 to assess its added predictive value. PModel 4 further adjusts for estimated immune cell composition, providing a sensitivity analysis that accounts for variation in cell type proportions. PModel 5 further includes baseline waist circumference to evaluate whether predictive performance is independent of central adiposity.

**Table 4:** Predictive Performance of the Models in the Independent Testing Set.

	AUC	Accuracy	Sensitivity	Specificity	Precision	NPV	F1	Brier Score	Log Loss
PModel 1	0.398	0.500	0.875	0.050	0.525	0.250	0.656	0.275	0.750
PModel 2	0.652	0.614	0.583	0.650	0.667	0.565	0.622	0.236	0.661
PModel 3	0.625	0.591	0.542	0.650	0.650	0.542	0.591	0.250	0.693
PModel 4	0.596	0.523	0.458	0.600	0.579	0.480	0.512	0.268	0.742
PModel 5	0.644	0.545	0.542	0.550	0.591	0.500	0.565	0.248	0.712

All performance metrics were evaluated in the independent testing set. Accuracy, sensitivity, specificity, precision, negative predictive value (NPV), and F1 score were calculated using a probability threshold of 0.50. Lower Brier scores and log-loss values indicate better probabilistic prediction. PModel 1 includes baseline covariates: SES, smoking status, chronological age, and the time interval between study visits. PModel 2 includes only the standardized MRS. PModel 3 combines the MRS with the PModel 1 covariates. PModel 4 further adjusts for estimated immune cell composition to account for variation in blood cell type proportions. PModel 5 combines the MRS and baseline covariates from PModel 3 with baseline waist circumference to evaluate whether predictive performance is independent of central adiposity.

**Table 5:** Summary of Previous Studies Reported Associations Between CpG Loci and Metabolic Syndrome (MetS) or MetS-related components and results of this study in the a priori analysis.

Gene Name	Probe ID	Direction of the Association with MetS *		Reference
		This Study	Previous Studies	
*CPT1A*	cg00574958	−	−	Das et al., 2016 [3];
	cg17058475	−	−	Das et al., 2016 [3]
*SOCS3*	cg18181703	+	−	Ali et al., 2016 [10]
*SREBF1*	cg11024682	++	+	Hidalgo et al., 2021 [11]; Nuotio et al., 2020 [16]
*TXN1P*	cg19693031	−	−	Nuotio et al., 2020 [16]
*ABCG1*	cg06500161	+	+	Akinyemiju et al., 2018 [5]; Nuotio et al., 2020 [16];
	cg27243685	+	+	Yoon et al., 2017 [12]
*PHOSPHO1*	cg02650017	−−	+/−	Hidalgo et al., 2021 [11];
			−	Jones et al., 2023 [17];
*HIF3A*	cg22891070	++	+ ^‡^	Dick et al. 2014 [18]

* The direction of association with MetS is indicated as “+” for a positive association and “−” for a negative association. Double + or − indicates statistically significant in our study. The “+/−” designation reflects inconsistent or mixed findings across studies. ^‡^ Previous studies of *HIF3A* cg22891070 have primarily reported associations with MetS-related traits, including BMI, adiposity, body fat distribution, insulin sensitivity, and other obesity-related metabolic phenotypes, rather than MetS as a composite outcome.

**Table 6:** Selected CpG Loci.

Probe ID	Gene Name	Function
cg00574958	*CPT1A*	Involved in breaking down fats and converting them into energy in the liver
cg17058475		
cg18181703	*SOCS3*	Important for immune balance, and its dysfunction can lead to diseases like allergy, autoimmune disorders, and cancer
cg11024682	*SREBF1*	Plays a key role in the induction of lipogenesis by the liver
cg19693031	*TXN1P*	Plays an essential role in metabolism maintenance by activating inflammatory signaling
cg06500161	*ABCG1*	Controls lipoprotein lipase activity and promotes lipid accumulation in human macrophages in the presence of triglyceride-rich lipoproteins
cg27243685		
cg06638433	*IGF2BP1*	Post-transcriptional regulator, part of the insulin-like growth factor 2 mRNA-binding protein family
cg24876164		
cg02650017	*PHOSPHO1*	Plays a critical role in vascular smooth muscle cell mineralization
cg22891070	*HIF3A*	Low expression may adversely affect whole-body insulin sensitivity

## Data Availability

Data described in the manuscript will be made available on request to KEP and MMTR, pending review and approval by the ELEMENT Executive Committee.

## Supplementary Materials

The following supporting information can be downloaded at https://www.mdpi.com/article/10.3390/epigenomes10040060/s1, Table S1: Changes in clinical measurements from baseline to follow-up stratified by follow-up metabolic syndrome status; Table S2: Odds ratios (ORs) for the associations between DNA methylation at selected CpG loci and MetS; Table S3: Comparison of logistic regression models with and without adjustment for baseline waist circumference; Table S4: Associations of DNA Methylation with MetS in an Epigenome-Wide Association Study (EWAS); Table S5: Associations of DNA Methylation with Elevated Triglycerides (Epigenome-Wide Association Study); Table S6: Associations of DNA Methylation with Elevated Blood Pressure (Epigenome-Wide Association Study); Table S7: Associations of DNA Methylation with HDL Cholesterol (Epigenome-Wide Association Study); Table S8: Associations of DNA Methylation with Fasting Glucose (Epigenome-Wide Association Study); Table S9: Association between standardized MRS and MetS across logistic regression models in the training set.

## Abbreviations

The following abbreviations are used in this manuscript:

AMAI	Asociación Mexicana de Agencias de Investigación de Mercado y Opinión Pública
AUC	area under the curve
BMI	body mass index
CpG	cytosine–phosphate–guanine
CVD	cardiovascular disease
ELEMENT	Early Life Exposure in Mexico to Environmental Toxicants
EWAS	epigenome-wide association study
HDL	high density lipoprotein
MetS	metabolic syndrome
MRS	methylation risk score
MSE	mean squared error
OR	odds ratio
ROC	receiver operating characteristic
SD	standard deviation
SES	socioeconomic status
T2D	type II diabetes
