# Supplementary material for: DNA Methylation and Metabolic Syndrome in Midlife Women of the ELEMENT Cohort
Source: Epigenomes. 2026 Sep 24;10(4):60. doi: 10.3390/epigenomes10040060 (PMC13692396; doi:10.3390/epigenomes10040060)
Supplement: Supplementary file 1 [file epigenomes-10-00060-s001.zip › epigenomes-4576622-supplementary.pdf]

Supplementary Information

DNA Methylation and Metabolic Syndrome in Midlife Women of the ELEMENT Cohort

Authors:
Jiahe Tian [1], Jaclyn M. Goodrich [1,*], Lindsey English [2], Dana C. Dolinoy [1,2], Erica C. Jansen [2], Peter X. K. Song [3], Libni A. Torres-Olascoaga [4], Martha M. Téllez-Rojo [4], Karen E. Peterson [1,2]

Affiliations:
[1] Department of Environmental Health Sciences, University of Michigan School of Public Health, Ann Arbor, MI, USA; jhtian@umich.edu (J.T.); ddolinoy@umich.edu (D.C.D.); karenep@umich.edu (K.E.P.)
[2] Department of Nutritional Sciences, University of Michigan School of Public Health, Ann Arbor, MI, USA; lindseae@umich.edu (L.E.); janerica@umich.edu (E.C.J.)
[3] Department of Biostatistics, University of Michigan School of Public Health, Ann Arbor, MI, USA; pxsong@umich.edu
[4] National Institute of Public Health, Cuernavaca, Mexico; libniavib@gmail.com (L.A.T.-O.); mmtellez@insp.mx (M.M.T.-R.)

**Address correspondence to*:
1415 Washington Heights
Ann Arbor, MI 48109 USA
Telephone: 17346474564
Email: gaydojac@umich.edu

Table of Contents

Table S1 Changes in clinical measurements from baseline to follow-up stratified by follow-up metabolic syndrome status 3
Table S2. Odds ratios (OR) for the associations between DNA methylation at selected CpG loci and MetS. 4
Table S3 Comparison of logistic regression models with and without adjustment for baseline waist circumference 5
Table S4. Associations of DNA Methylation with MetS in an Epigenome-Wide Association Study (EWAS). 6
Table S5. Associations of DNA methylation with Elevated Triglycerides (Epigenome-Wide Association Study). 8
Table S6. Associations of DNA methylation with Elevated Blood Pressure (Epigenome-Wide Association Study). 9
Table S7. Associations of DNA methylation with HDL Cholesterol (Epigenome-Wide Association Study). 11
Table S8. Associations of DNA methylation with Fasting Glucose (Epigenome-Wide Association Study). 12
Table S9. Association between standardized MRS and MetS across logistic regression models in the training set. 13

Table S1 Changes in clinical measurements from baseline to follow-up stratified by follow-up metabolic syndrome status

Variable	MetS status	N	Baseline Mean (SD)	Follow-up Mean (SD)	Mean change	Within-group P
**BMI (kg/m²)**	MetS−	62	27.61 (4.57)	28.93 (4.36)	1.32	<0.001
	MetS+	87	29.86 (4.84)	31.35 (4.89)	1.49	<0.001
**Systolic BP (mmHg)**	MetS−	62	103.19 (11.51)	111.81 (12.84)	8.61	<0.001
	MetS+	87	110.05 (11.68)	121.18 (18.65)	11.14	<0.001
**Diastolic BP (mmHg)**	MetS−	62	68.05 (9.25)	71.85 (7.32)	3.81	0.008
	MetS+	87	72.36 (7.84)	76.02 (10.21)	3.67	0.003
**Waist circumference (cm)**	MetS−	62	84.23 (9.80)	97.66 (9.61)	13.43	<0.001
	MetS+	87	89.37 (10.08)	104.17 (11.34)	14.81	<0.001

Values are presented as mean (standard deviation). Mean change was calculated as the follow-up value minus the baseline value. Within-group P values were calculated using paired t-tests among participants with complete measurements at both visits. MetS status was determined at the follow-up visit.

Table S2. Odds ratios (OR) for the associations between DNA methylation at selected CpG loci and MetS.
Results from the logistic regression models assessing prospective associations between DNA methylation at *a priori* loci and Metabolic Syndrome in ELEMENT cohort women.

		**Logistic Regression**							
		**Model 1**		**Model 2**		**Model 3**		**Model 4**	
Gene	CpG loci	*P*-value	OR (95% CI)	*P*-value	OR (95% CI)	*P*-value	OR (95% CI)	*P*-value	OR (95% CI)
TXNIP	cg19693031	0.112	0.94 (0.88, 1.01)	0.074	0.93 (0.87, 1.01)	0.077	0.93 (0.87, 1.01)	0.119	0.94 (0.87, 1.02)
CPT1A	cg00574958	0.452	0.95 (0.84, 1.08)	0.359	0.94 (0.82, 1.07)	0.338	0.94 (0.82, 1.07)	0.474	0.95 (0.83, 1.09)
CPT1A	cg17058475	0.570	0.96 (0.84, 1.10)	0.083	0.86 (0.73, 1.02)	0.083	0.86 (0.73, 1.02)	0.103	0.87 (0.73, 1.03)
SREBF1	cg11024682	**<0.001**	**1.32 (1.15, 1.52)**	**<0.001**	**1.33 (1.14, 1.54)**	**<0.001**	**1.33 (1.14, 1.55)**	**0.001**	**1.29 (1.11, 1.51)**
IGF2BP1	cg06638433	0.790	1.01 (0.91, 1.13)	0.877	1.01 (0.90, 1.14)	0.891	1.01 (0.90, 1.13)	0.987	1.00 (0.88, 1.13)
IGF2BP1	cg24876164	0.375	0.93 (0.79, 1.09)	0.317	0.92 (0.78, 1.08)	0.321	0.92 (0.78, 1.09)	0.455	0.94 (0.79, 1.11)
PHOSPHO1	cg02650017	**0.007**	**0.76 (0.62, 0.93)**	**0.024**	**0.78 (0.62, 0.97)**	**0.025**	**0.78 (0.62, 0.97)**	0.074	0.81 (0.64, 1.02)
SOCS3	cg18181703	0.392	1.03 (0.96, 1.12)	0.236	1.05 (0.97, 1.14)	0.249	1.05 (0.97, 1.14)	0.212	1.05 (0.97, 1.15)
HIF3A	cg22891070	**0.005**	**1.04 (1.01, 1.07)**	**0.006**	**1.04 (1.01, 1.07)**	**0.005**	**1.04 (1.01, 1.07)**	**0.008**	**1.04 (1.01, 1.07)**
ABCG1	cg27243685	0.547	1.04 (0.91, 1.20)	0.641	0.96 (0.82, 1.13)	0.658	0.96 (0.82, 1.14)	0.761	0.97 (0.82, 1.16)
ABCG1	cg06500161	0.076	1.13 (0.99, 1.30)	**0.046**	**1.16 (1.00, 1.34)**	**0.048**	**1.16 (1.00, 1.34)**	0.171	1.11 (0.95, 1.30)

Odds ratios and 95% confidence intervals are reported per 1-percentage-point increase in DNA methylation. Model 1 adjusted for smoking status, maternal age, and socioeconomic status. Model 2 additionally adjusted for estimated blood cell proportions. Model 3 additionally adjusted for the time between DNA methylation measurement and metabolic syndrome assessment. Model 4 additionally adjusted for baseline waist circumference. Bold values indicate P < 0.05.

Table S3 Comparison of logistic regression models with and without adjustment for baseline waist circumference

Gene	CpG loci	β Model 3	β Model 4	% Change in β	AIC Model 3	AIC Model 4	ΔAIC	LRT *P*-value
TXNIP	cg19693031	-0.068	-0.063	7.5	220.14	211.18	-8.97	<0.001
CPT1A	cg00574958	-0.065	-0.051	21.5	222.63	213.30	-9.34	<0.001
CPT1A	cg17058475	-0.146	-0.144	1.5	220.46	211.10	-9.36	<0.001
SREBF1	cg11024682	0.284	0.257	9.5	207.40	201.57	-5.82	0.005
IGF2BP1	cg06638433	0.008	0.001	87.0	223.53	213.81	-9.72	<0.001
IGF2BP1	cg24876164	-0.084	-0.065	23.1	222.50	213.22	-9.28	<0.001
PHOSPHO1	cg02650017	-0.253	-0.212	16.4	218.19	210.48	-7.71	0.002
SOCS3	cg18181703	0.047	0.053	-12.7	222.21	212.23	-9.98	<0.001
HIF3A	cg22891070	0.040	0.039	1.5	215.13	206.28	-8.85	<0.001
ABCG1	cg27243685	-0.037	-0.027	27.6	223.35	213.71	-9.64	<0.001
ABCG1	cg06500161	0.148	0.108	27.1	219.48	211.91	-7.57	0.002

Model 3 adjusted for smoking status, age, socioeconomic status, estimated blood cell proportions, and time between visits. Model 4 additionally adjusted for baseline waist circumference. % change in β was calculated as (βModel 3 − βModel 4) / βModel 3 × 100. Positive values indicate attenuation of the CpG effect estimate after adjustment for baseline waist circumference. ΔAIC was calculated as AICModel 4 − AICModel 3. Negative values indicate a lower AIC after adjustment for baseline waist circumference. The likelihood ratio test compares Model 3 and Model 4, with Model 3 nested within Model 4.

Table S4. Associations of DNA Methylation with MetS in an Epigenome-Wide Association Study (EWAS).
Results from the logistic regression models assessing prospective associations between DNA methylation at all loci on the EPIC array passing quality control and Metabolic Syndrome in ELEMENT cohort women. Loci with p-value< 1.0×10^{-4} are shown.

CpG ID	Gene	Chromosome	Position	OR (95% CI)	Related traits
cg25375589	NA	chr3	43220779	1.34 (1.18, 1.52)	Smoking
cg09276573	*PLCH1*	chr3	155394092	0.62 (0.51, 0.77)	Differentiation of skeletal muscle
cg22595391	*C17orf62*	chr17	80407031	0.5 (0.37, 0.69)	Psoriasis
cg13929323	NA	chr9	114364066	1.16 (1.08, 1.25)	**Aging**
cg13538156	*MAGEB1*	chrX	30261568	0.78 (0.7, 0.88)	Adenoma
cg14627781	*KPNA5*	chr6	117062904	1.17 (1.09, 1.26)	Smoking
cg10123945	NA	chr4	74680719	0.54 (0.4, 0.72)	NA
cg24941195	*C7orf65*	chr7	47694816	0.68 (0.57, 0.82)	Air pollution
cg12680737	*KLRC1*	chr12	10607782	0.76 (0.67, 0.87)	NA
cg08242636	*CBFB*	chr16	67062389	1.78 (1.35, 2.35)	**T2D; Obesity**
cg03692519	*GALNT18*	chr11	11591736	1.3 (1.15, 1.48)	NA
cg09803392	NA	chr11	130659818	0.67 (0.56, 0.82)	NA
cg16438303	*POLR2C*	chr16	57496824	0.29 (0.16, 0.53)	NA
cg08252325	NA	chr2	71098940	0.21 (0.1, 0.45)	NA
cg05713794	*GP5*	chr3	194119861	1.44 (1.2, 1.72)	Down syndrome
cg00693776	*NEBL*	chr10	21105179	1.29 (1.14, 1.45)	NA
cg03440711	NA	chr2	218040346	0.53 (0.39, 0.72)	NA
cg09320591	*LINC00403*	chr13	112761361	1.26 (1.13, 1.42)	NA
cg01883759	*JAZF1*	chr7	28220576	0.71 (0.6, 0.84)	Preterm birth; birth weight
cg17668115	*PEAR1*	chr1	156868625	1.23 (1.11, 1.37)	Lung cancer
cg21120661	*TRPM2*	chr21	45810508	0.68 (0.57, 0.83)	Asthma
cg01652542	*RALY-AS1*	chr20	32581446	0.16 (0.06, 0.4)	NA
cg20716774	*ANKRD33B*	chr5	10601638	1.32 (1.15, 1.51)	NA

cg12742338	*CLIC6*	chr21	36042464	0.89 (0.84, 0.94)	Colorectal cancer
cg11270792	NA	chr1	156586531	1.33 (1.15, 1.53)	NA
cg11024682	*SREBF1*	chr17	17730094	1.31 (1.15, 1.5)	**MetS; T2D; waist circumference**
cg05518088	NA	chr3	34463554	1.16 (1.08, 1.25)	Alzheimer's Disease
cg09898729	*C1orf212*	chr1	35325189	0.37 (0.23, 0.61)	Gestational diabetes mellitus
cg07955965	*ZNRD1*	chr6	30029370	0.2 (0.09, 0.45)	NA
cg05997357	*NCOA2*	chr8	71064794	0.81 (0.73, 0.9)	NA
cg16536807	NA	chr2	204490670	0.52 (0.38, 0.72)	NA
cg08827579	*AKNA*	chr9	117150458	0.35 (0.21, 0.59)	NA
cg15539073	*ZNF536*	chr19	30897675	1.33 (1.15, 1.54)	B Acute Lymphoblastic Leukemia

* NA: Not Applicable: no gene name because it is in an intergenic region or no related traits on the EWAS Atlas website; T2D: type 2 diabetes

Table S5. Associations of DNA methylation with Elevated Triglycerides (Epigenome-Wide Association Study).
Results from the logistic regression models assessing prospective associations between DNA methylation at all loci on the EPIC array passing quality control and high triglycerides in ELEMENT cohort women. Loci with p-value< 1.0×10^{-4} are shown.

CpG ID	Gene	Chromosome	Position	OR (95% CI)	Related traits
cg06529416	NA	chr10	102381881	0.8 (0.73, 0.89)	NA
cg03437318	*ERBB3*	chr12	56474175	0.5 (0.36, 0.69)	**Aging**
cg05997357	*NCOA2*	chr8	71064794	0.79 (0.7, 0.88)	**Aging**
cg27355354	*C7orf51*	chr7	100081519	0.04 (0.01, 0.19)	**Obesity**
cg02084758	NA	chr17	77536754	22.3 (4.91, 101.19)	NA
cg25963429	*CREB3L1*	chr11	46301410	1.41 (1.19, 1.68)	Psoriasis
cg15860624	*ZFR2*	chr19	3811194	1.22 (1.1, 1.34)	**Hepatic fat**
cg23775011	*UST*	chr6	149068387	0.47 (0.32, 0.68)	NA
cg17592376	*PTPRG*	chr3	61828811	0.64 (0.51, 0.8)	**Aging**
cg12074247	NA	chr13	34675978	0.72 (0.61, 0.85)	NA
cg24550676	*DND1*	chr5	140053903	1.31 (1.15, 1.5)	Down Syndrome

* NA: Not Applicable, no gene name because it is in an intergenic region or no related traits on the EWAS Atlas website.

Table S6. Associations of DNA methylation with Elevated Blood Pressure (Epigenome-Wide Association Study).
Results from the logistic regression models assessing prospective associations between DNA methylation at all loci on the EPIC array passing quality control and high blood pressure in ELEMENT cohort women. Loci with p-value< 1.0×10^{-4} are shown.

CpG ID	Gene	Chromosome	Position	OR (95% CI)	Related traits
cg19639533	NA	chr8	144870260	0.36 (0.23, 0.56)	Breast cancer prognosis
cg10519140	*SLC29A1*	chr6	44187076	2.48 (1.65, 3.71)	Immune and developmental regulation functions
cg06819923	*ZP2*	chr16	21214508	1.35 (1.17, 1.56)	**Aging**
cg08141163	*COQ6*	chr14	74425145	0.27 (0.15, 0.51)	**Weight loss**
cg21195235	*KATNAL2*	chr18	44530218	1.34 (1.16, 1.54)	Thyroid lesion
cg06270361	*DEDD*	chr1	161100993	0.44 (0.3, 0.66)	**Obesity**
cg01323801	NA	chr16	3692200	0.57 (0.43, 0.75)	NA
cg01204915	NA	chr4	87777194	0.24 (0.12, 0.48)	NA
cg20463817	NA	chr9	82975989	0.4 (0.25, 0.62)	NA
cg22183057	*NUDCD2*	chr5	162883763	0.26 (0.13, 0.5)	Rheumatoid arthritis
cg13904574	*CNTFR*	chr9	34586050	1.34 (1.16, 1.55)	**Aging, Atherosclerosis**
cg01111414	NA	chr7	33711078	1.36 (1.17, 1.59)	NA
cg04348086	*ATP2B2*	chr3	10502737	0.76 (0.66, 0.87)	**Aging, Obesity, T2D**
cg14812930	*PARP12*	chr7	139724757	0.5 (0.35, 0.7)	**Aging**
cg07795447	NA	chr10	3446963	0.41 (0.26, 0.63)	NA
cg12586496	*DNASE2*	chr19	12992070	15.3 (3.99, 58.69)	**Aging, Dilated Cardiomyopathy**
cg07266837	NA	chr16	75555472	1.38 (1.18, 1.61)	NA
cg16502517	*ALS2CL*	chr3	46718727	0.56 (0.42, 0.75)	Breast cancer prognosis
cg12519228	*GNB5*	chr15	52454148	0.46 (0.32, 0.68)	NA
cg11845761	*LOC100287944*	chr12	107161891	1.33 (1.16, 1.54)	NA
cg15879261	*PALLD*	chr4	169598279	0.42 (0.27, 0.64)	**Aging**
cg07130293	*PKIG*	chr20	43220539	0.35 (0.21, 0.59)	NA
cg10373024	*DDX54*	chr12	113603735	0.49 (0.34, 0.7)	Bariatric surgery
cg09035065	*TNS1*	chr2	218809808	0.28 (0.15, 0.53)	**Aging**
cg22174399	*STAG1*	chr3	136265122	0.47 (0.32, 0.68)	Anthracycline-induced cardiomyopathy

cg23679073	*ZC3H15*	chr2	187350909	42.08 (6.51, 272.11)	NA
cg18404346	*BRF1*	chr14	105775930	0.49 (0.35, 0.7)	Breast cancer prognosis
cg06808571	*KCNH2*	chr7	150642256	1.29 (1.14, 1.47)	**Aging**
cg09750651	NA	chr11	134944070	0.57 (0.43, 0.75)	NA
cg25636224	*WNK2*	chr9	95947173	23.3 (4.82, 112.54)	Inflamed Crohn's disease

* NA: Not Applicable, no gene name because it is in an intergenic region or no related traits on the EWAS Atlas website; T2D: type 2 diabetes

Table S7. Associations of DNA methylation with HDL Cholesterol (Epigenome-Wide Association Study).
Results from the logistic regression models assessing prospective associations between DNA methylation at all loci on the EPIC array passing quality control and low HDL cholesterol in ELEMENT cohort women. Loci with p-value< 1.0×10^{-4} are shown.

CpG ID	Gene	Chromosome	Position	OR (95% CI)	Related traits
cg01736844	*ZNF483*	chr9	114286842	0.32 (0.19, 0.56)	Hepatocellular carcinoma
cg18804734	*CHAF1A*	chr19	4402429	0.32 (0.19, 0.56)	NA
cg25972591	*ZSCAN18*	chr19	58627987	0.49 (0.35, 0.7)	**Aging**
cg06046858	*HAPLN1*	chr5	82978409	0.46 (0.31, 0.67)	**Aging**
cg02944422	*ZNF81*	chrX	47696228	1.3 (1.14, 1.49)	**Aging**
cg01604994	*AGAP1*	chr2	236867836	0.58 (0.44, 0.76)	**Aging**

* NA: Not Applicable, no gene name because it is in an intergenic region or no related traits on the EWAS Atlas website.

Table S8. Associations of DNA methylation with Fasting Glucose (Epigenome-Wide Association Study).
Results from the logistic regression models assessing prospective associations between DNA methylation at all loci on the EPIC array passing quality control and elevated fasting glucose in ELEMENT cohort women. Loci with p-value< 1.0×10^{-4} are shown.

CpG ID	Gene	Chromosome	Position	OR (95% CI)	Related traits
cg19912283	*VARS2*	chr6	30884590	2.87 (1.77, 4.67)	**Aging**
cg21649965	NA	chr12	67555108	3.58 (1.94, 6.6)	NA
cg16834683	NA	chrX	39514130	0.78 (0.69, 0.88)	NA
cg05088257	*UBASH3B*	chr11	122621046	1.57 (1.26, 1.96)	**Aging, Mortality**
cg15102842	*ZNF407*	chr18	72369543	2.97 (1.74, 5.07)	NA
cg07053189	*AFTPH*	chr2	64779233	3.66 (1.93, 6.93)	NA
cg24530527	*C7orf50*	chr7	1162932	0.03 (0, 0.17)	Breast cancer prognosis

* NA: Not Applicable, no gene name because it is in an intergenic region or no related traits on the EWAS Atlas website.

Table S9. Association between standardized MRS and MetS across logistic regression models in the training set.

	OR (95% CI)	P-value
**PModel 2**	3.55 (2.03–6.22)	<0.001
**PModel 3**	3.82 (2.11–6.93)	<0.001
**PModel 4**	4.74 (2.30–9.76)	<0.001
**PModel 5**	4.41 (2.09–9.34)	<0.001

Odds ratios (ORs) represent the change in odds of MetS per 1-standard-deviation increase in the standardized MRS. Estimates were obtained from logistic regression models fitted in the complete 70% training set. PModel 2 includes only the standardized MRS. PModel 3 combines the MRS with baseline covariates: SES, smoking status, chronological age, and the time interval between study visits. PModel 4 further adjusts for estimated immune cell composition. PModel 5 additionally includes baseline waist circumference.
